# News and Notes

**Published:** 1900-06

**Authors:** 


### NEWS AND NOTES.

### Fame.

A leaf uptossed from a mimic whirlwind of leaves,
An earth-bound bird that, brain pierced, soars above the ultimate flight of the eagle,
The frothing crest of a strenuous wave of the shambling sea,
A firebrand shot whirling above the lurid heart of the conflagration,
The vibrant profundo of a thick-lipped gun's diapason.

The leaf settles down with the passing of the circular current,
The bird drops dead to earth at the end of its stricken delirium,
The wave is smoothed out in the ceaseless fling of the tide,
The firebrand, blackened and charred, falls a stick, and is swept in the rubbish,
The echo of the cannon is heard no more in the peaceful hills—
This is Fame. This also is Fame's evanescence.

Bernard Wolff in *The Raven.*

---

The estate left by Sir James Paget amounted to $370,000, which is divided almost equally among his six children.

---

Diphtheria is almost an unheard-of disease at Johannesburg. The reason for this is said to be that the elevation of this town is over 4,000 feet.

---

Drs. Robert F. Weir and Wm. T. Bull have been appointed to succeed Dr. McBurney, who recently resigned as chief surgeon of Roosevelt Hospital, New York.

---

The *Mediaal News* is authority for the statement that Mrs. Mary Baker G. Eddy, leader and founder of the "Christian Science" Church, is very ill at her home in Concord, N. H., with a cancerous affection.

---

A sewing needle, two and one-half inches long, was recently found in the vermiform appendix of a patient operated upon at

Hartford, Conn., for appendicitis. The young woman remembers having swallowed a needle several years ago.

---

DR. W. A. O'DANIEL of Bullard, Ga., died on May 22d. Dr. O'Daniel was one of the most prominent physicians in the State, besides being actively identified with State politics. He was always a working member of the Georgia Medical Asssociation, and did much to advance the interests of this body.

---

A FRENCH physician who has a very extensive country practice carries with him, the *Lyon Medical* says, several carrier-pigeons. When he finds a patient in urgent need of medicine he attaches the prescription under the wing and releases the bird. On the appearance of the latter, the apothecary catches it, and dispatches the remedy, thus frequently saving many hours' delay. The idea is an excellent one, which it might pay some other druggists and physicians who have long routes to adopt.

---

THE attorney-general of the State of Texas instituted *quo warranto* proceedings against the New York Medical College of San Antonio for forfeiture of charter, and to restrain the concern from issuing diplomas for the practice of medicine. This "college" was incorporated for the purpose of teaching medicine by mail or otherwise. Its diplomas in the State are valid in law, but the complainant avers that "the main object and practice of the defendant is to sell its diplomas to any person it pleases, whether informed or not in the science of medicine, for the stipulated sum of $50." Specific charges made. Good for Texas!—*N. O. Med. and Sur. Journal.*

---

**This Journal and Atlanta Semi-weekly Journal one year for $1.00 cash in advance. No checks. Strictly to new subscribers.**

THE Western Ophthalmologic and Oto-Laryngologic Association, at its fifth annual meeting held in St. Louis, April 5–7, 1900, elected the following officers: President, M. A. Goldstein, M.D., St. Louis, Mo.; First Vice-President, H. V. Würdemann, M.D., Milwaukee, Wis.; Second Vice-President, C. R. Holmes, M.D., Cincinnati, Ohio; Third Vice-President, F. C. Ewing, M.D., St. Louis, Mo.; Secretary, W. L. Ballenger, M.D., Chicago, Ill.; Treasurer, W. L. Dayton, M.D., Lincoln, Neb. The next meeting will be held in Cincinnati, Ohio.

---

**This Journal and Atlanta Semi-weekly Journal one year for $1.00 cash in advance. No checks. Strictly to new subscribers.**

---

ACCORDING to the *Medical Times and Register* for April, the London *Times* describes the visit of a celebrated doctor named Chen in medical attendance upon the emperor. He was commanded to appear, and 6,000 taels (about $4,500) was paid to him in advance for his traveling expenses and fee. He was told that the emperor was suffering in his organs of breathing, from feverishness and general weakness. He was not allowed to ask his patient a question, and, though admitted twice to his presence, he was compelled to cross the room upon his knees, keeping his eyes constantly upon the floor. The empress described to the doctor the patient's symptoms, but he was not allowed to feel his pulse, though he was permitted to lay his flat hand upon the person of his sovereign. Dr. Chen is said to have remarked that, under these circumstances, one doctor was as good as another, and he petitioned that he might be allowed to return home on account of the sickness of his aged mother. This form of excuse being very common, the matter was inquired into, and Dr. Chen was able, by expending 18,000 taels, to prove that he had in fact an aged mother, and that she was sick, whereupon permission was given him to return home. His escape may have been the means of saving his life, but it cost him a good deal of money.—*N. Y. Medical Journal.*

---

***NOTICE.—This JOURNAL and THE INTERNATIONAL JOURNAL OF SURGERY one year for $1.00 cash, to new subscribers. Out-of-town checks not accepted unless 10c. is added for cost of collection. Strictly to new subscribers.***

---

TWO YEARS ago at the meeting of the Confederate veterans in Atlanta the Association of the Medical Officers of the Confederate Army and Navy was organized. During the coming Confederate reunion in this city on May 30th this Association will convene for the double purpose of renewing the bonds of comradeship formed in the early sixties and furthering its organization. The Medical Committee has issued a handsome card of invitation to every physician whose name and address it could command who was a medical officer, a veteran or a son of a veteran. The response has been very gratifying to those in charge of the medical department, and it is expected that the attendance will exceed that of any previous meeting.

The present officers of the Association are: Dr. Francis L. Parker, of Charleston, S. C., President; Dr. J. J. Knott, of Atlanta, Ga., Vice-President; Dr. V. G. Hitt, of Atlanta, Ga., Secretary and Treasurer; Rev. A. T. Porter, D.D., of Charleston, S. C., Chaplain. At this year's meeting new officers will be elected to serve for the ensuing year. Several interesting papers on the military surgery of the civil war are expected to be read before the Association.

The local committee, with Dr. Preston B. Scott as chairman, has spared no effort to make the reunion a success, and the entertainment of the visiting veterans has been fully provided for. The daily meetings of the Scottish Rite Cathedral will partake much of a social nature. The physicians of the city are expected to meet the Association at these times and contribute their quota toward extending the hospitality of the city to its visitors. At these meetings a luncheon will be served by the wives of the physicians of the city.

The record of the medical staff of the Confederate army is a memorable one, and one to which its survivors may well point with pride. Handicapped by the edict of the Federal Government declaring medicines and surgical appliances contraband of war, they waged a most remarkable battle against disease and death in the camps and prisons under their jurisdiction. The small percentum of losses of sick and wounded in their care stands almost alone in the history of civilized warfare.

With the flood of happy memories conjured up from behind a veil of almost forty years by each hand-clasp, there rises a tinge of sadness. Year by year the muster-roll of those who gave their services to the cause of "the bonnie blue flag" has dwindled. Each year, from the great silent camps of the soldier dead, comrades have hailed comrades with a last call of their battered bugles, and each year the echo of "taps" has sounded deeper and louder in the hearts of the living; yet no longer with the blare of sounding brass. For the notes are framed by the dead lips of comrades, and the sound has an ineffable sweetness.

Men of the South, we give you welcome. In behalf of the physicians of our city we extend to you the right hand of good fellowship and best wishes for a successful meeting and a prosperous future.—*The American Practitioner and News.*

